# Global Surgery – Redirecting Strategies for a Global Research Agenda: Comment on "Global Surgery – Informing National Strategies for Scaling Up Surgery in Sub-Saharan Africa"

**DOI:** 10.15171/ijhpm.2018.79

**Published:** 2018-08-25

**Authors:** Jaymie A. Henry

**Affiliations:** Department of Surgery, Charles E. Schmidt College of Medicine, Florida Atlantic University, Boca Raton, FL, USA.

**Keywords:** Global Surgery, Essential Surgery, Universal Health Coverage, WHO Surgical Resolution

## Abstract

More than three years have passed since the publication of the Lancet Commission on Global Surgery and its recommendations on scaling up surgery in sub-Saharan Africa (SSA). An important gap, the voice of the districts as well as lack of contextualized research, has been noted in its support of national surgical plans that run the risk of being at best, aspirational. Moreover, a ‘one-size-fits-all approach’ may not adequately address country-specific challenges on the ground. There is a need to redirect attention, effort, and funding in creating a global mechanism to gather baseline country information documenting every single district level government health facility’s ability and readiness to provide safe surgical, obstetric, trauma, and anesthesia care using the World Health Organization (WHO) Service Availability and Readiness Assessment (SARA) tool to aid in directing country-specific efforts in surgical systems strengthening and ensuring that a basic package of essential surgical and anesthesia services is made available to each citizen with adequate financial protection by 2030. This global mechanism will enable benchmarking, accountability, and streamlining of the work of the global surgical community to achieve true progress in scaling up surgery not only in SSA, but for the rest of the developing world.

The accompanying article by Gajewski et al^1^ is a critique of the progress made in the three years since the publication of the Lancet Commission on Global Surgery and of efforts in scaling up essential surgical care in sub-Saharan Africa (SSA). The authors outlined the recommendations set forth by the group as well as describe the efforts of the Harvard Program in Global Surgery and Social Change to support the development of the national surgical plan of Zambia through reviews of national level data, semi-structured interviews of country specialists, and writing workshops. They also point out that while the design process has been consultative, the major weakness lies in its lack of contextualized research findings to support actual implementation and runs the risk of remaining at best, aspirational. Moreover, the exclusion of the voice of the districts where the need is greatest and where the bulk of the work lies is a real risk in underestimating the extent of the work that needs to be done. Modeling data on disease burden, cost and cost-effectiveness of surgical procedures and effectiveness of strategies to mitigate neglected surgical diseases to raise awareness, start the conversation at the global level, and to keep countries informed of benchmarks is invaluable, and have been effective in doing so. One must not overvalue its utility; however, as countries still need baseline information from which to gather valuable insight to inform their priorities moving forward. Thus, the authors’ recommendation to focus on surgical systems research that includes district level data, is well founded. The authors mention the dearth of empirical research on surgical capacity at district-level hospitals (DLHs), which may be accurate considering the fact that the 21 published surgical capacity studies in 17 low- and middle-income countries (LMICs) yielded only seven additional studies with any combination of previous studies with analysis going beyond establishing a baseline.^2^ Moreover, different tools were utilized in assessing baseline surgical capacity, rendering impossible the formation of any kind of regional or global data that can reliably inform policymakers. Hence, there is a need to develop tools that can be adapted in a contextually relevant manner for baseline assessment and monitoring of the process.

We still face a lack of information on the impact of interventions that can reliably scale global surgical care.^1^ As the paper rightly points out, there is a need to coordinate research initiatives to inform the global surgical community on the feasibility, impact, and cost-effectiveness of scalable surgical interventions.

While increasing attention to assist countries in creating national surgical plans is commendable, several points deserve mention based on lessons learned in past experiences:

Expert published recommendations are useful; but needs a targeted advocacy strategy to inform policy-makers who may or may not be cognizant of the academic debates taking place. Moreover, demonstration of support from credible constituent bodies involved in implementation is crucial to getting their attention, engagement, and support. Indeed, surgical indicators in the World Health Organization (WHO), World Bank, and the United States Agency for International Development (USAID) Core 100 health indicators were only included after a targeted campaign by the G4 Alliance involving the support of 120 surgical, obstetric, trauma, and anaesthesia care organizations.^3^ The inclusion of surgical indicators at the World Development Indicators stemmed from the same effort. The landmark WHO Resolution WHA 68.15 on ‘Strengthening Emergency and Essential Surgical Care as a component of Universal Health Coverage’^4^ was the result of years of work with multiple layers of on the ground advocacy from multiple players with various government relationships.^5^ Without serious acknowledgement of their role, ‘Global Surgery’ will remain but an academic exercise.

Countries’ attention and interest in national surgical scale-up was stimulated by the WHO Resolution,^4^ but unless real funding has been committed, remains just an interest. To date, no country has committed dedicated funding to national surgical scale-up. Moreover, it is important to remember that countries can only be held accountable to the contents of the resolution and should be referred to often in national surgical planning efforts. Efforts to assist countries in writing national surgical plans is highly commendable, and have provided the impetus for greater political engagement. This strategy can be further strengthened by providing assistance in either conducting or financing national surgical capacity studies and providing technical support, pilot studies that aim to identify the most cost-efficient and effective strategy to scale-up surgical services, training and working with students and researchers from developing countries, funding global surgical initiatives that show promise, working with other research bodies to expand the global surgical research agenda, and helping to supply good quality data to groups such as the WHO GIEESC, and the G4 Alliance to aid in global advocacy. Without outputs that benefit the country such as baseline information to inform policymakers or validated pilot studies that demonstrate a successful program, the engagement from developed country research actors serve to benefit its own program more than the country itself as well as draw resources from the country by way of lost time to focus on more impactful activities such as implementation and training to build on the ground capacity. Moreover, the blueprint outlined as a result of an exhaustive consultative process by experts; while tremendously commendable, should be validated on the ground prior to being widely implemented. This avoids pitfalls from ‘Ivory Tower’ thinking without taking into account the highly complex nature of developing world challenges.

Data exist on the cost-effectiveness of certain interventions^6^ that can be provided at the district level.^7^ This can form the basis of a basic package of essential surgery and anesthesia services at the primary and first-referral hospital, which can be pilot-tested and scaled up systematically.

We should avoid the ‘Northern partners’ framework, as mentioned in the article, and should refrain from taking the lead in surgical development efforts and thus, taking agency from the developing countries themselves to develop their own strategies. Instead, we should facilitate South-South intersectoral collaboration, drive funding to grassroots initiatives that show success, and focus on translating high impact, low-cost technologies as a challenge to resource constrained areas of the world. Being a novel field, we can benefit from lessons learned in past global initiatives and avoid uncomfortable scenarios such as latrines built in developing countries that were never used,^8^ learn the nuances of why the widely distributed insecticide-treated malaria bed nets, while useful in combating the disease, had an unintended consequence on being used in fisheries,^9^ and health facilities that are equipped, but not staffed, and vice versa.^10^

It has been three and a half years since the WHO resolution was passed and only a handful of countries show some activity with no demonstrated impact on health outcomes such as maternal mortality rate and injury mortality rate, to name a few. Progress has been slow with no clear path to achieving WHA 68.15: ‘Strengthening Emergency and Essential Surgical Care as a component of Universal Health Coverage.’ I would like to therefore challenge the global surgical community: can we, as a group, once again unite for the neglected surgical patient, and come together in a serious effort to achieve universal health coverage? Can we establish a global mechanism to gather baseline country information documenting every single district level government health facility’s ability and readiness to provide safe surgical, obstetric, trauma, and anesthesia care using the WHO’s Service Availability and Readiness Assessment (SARA) tool as reported in a recent study^11^ to aid in ensuring that a basic package of surgical services is made available to each citizen with adequate financial protection by 2030? Can a large policy group with a track record of success step up to provide technical assistance in helping this massive effort get off the ground? The amount of work lies ahead, and it is without question enormous, and only with continued, dedicated, transparent, inclusive, and coordinated effort will the global surgical community actually, truly, scale-up surgical care that not only belongs to the annals but also to the neglected surgical patient, families, and societies on the ground.

## Ethical issues

Not applicable.

## Competing interests

Author declares that she has no competing interests.

## Author’s contribution

JAH is the single author of the paper.
